# Blind Quality Prediction for View Synthesis Based on Heterogeneous Distortion Perception

**DOI:** 10.3390/s22187081

**Published:** 2022-09-19

**Authors:** Haozhi Shi, Lanmei Wang, Guibao Wang

**Affiliations:** 1 School of Physics, Xidian University, Xi’an 710071, China; 2 School of Physics and Telecommunication Engineering, Shaanxi University of Technology, Hanzhong 723001, China

**Keywords:** view synthesis, quality prediction, two-channel convolutional neural network, heterogeneous distortion perception

## Abstract

The quality of synthesized images directly affects the practical application of virtual view synthesis technology, which typically uses a depth-image-based rendering (DIBR) algorithm to generate a new viewpoint based on texture and depth images. Current view synthesis quality metrics commonly evaluate the quality of DIBR-synthesized images, where the DIBR process is computationally expensive and time-consuming. In addition, the existing view synthesis quality metrics cannot achieve robustness due to the shallow hand-crafted features. To avoid the complicated DIBR process and learn more efficient features, this paper presents a blind quality prediction model for view synthesis based on HEterogeneous DIstortion Perception, dubbed HEDIP, which predicts the image quality of view synthesis from texture and depth images. Specifically, the texture and depth images are first fused based on discrete cosine transform to simulate the distortion of view synthesis images, and then the spatial and gradient domain features are extracted in a Two-Channel Convolutional Neural Network (TCCNN). Finally, a fully connected layer maps the extracted features to a quality score. Notably, the ground-truth score of the source image cannot effectively represent the labels of each image patch during training due to the presence of local distortions in view synthesis image. So, we design a Heterogeneous Distortion Perception (HDP) module to provide effective training labels for each image patch. Experiments show that with the help of the HDP module, the proposed model can effectively predict the quality of view synthesis. Experimental results demonstrate the effectiveness of the proposed model.

## 1. Introduction

With the wide application of multi-view video and free-view television, virtual view synthesis technology has developed rapidly [1,2]. The virtual multi-view synthesis technology interacts with texture and depth images from different viewpoints to generate new viewpoints, of which the depth-image-based rendering (DIBR) algorithm is the most commonly used and recognized [3,4]. In practice, distortions may occur in the acquisition, compression, and transmission of texture and depth images, which affect the image quality of view synthesis [5]. As a result, it is necessary to give a corresponding quality evaluation to measure and optimize the effect of view synthesis [6].

Image quality assessment (IQA) is divided into full-reference (FR), reduced-reference (RR), and no-reference (NR) [7]. FR-IQA methods require reference to the original distortion-free image for scoring. Typical FR-IQA methods include Structural Similarity (SSIM) [8], Information Fidelity Criterion (IFC) [9], and Visual Information Fidelity (VIF) [10]. RR-IQA methods use only a small amount of edge information extracted from the original distortion-free image as a reference for scoring [11,12]. However, in practical applications, the original image of the distorted image rarely exists. Hence, it is more practical to use the NR-IQA method, which does not require any information from the original distortion-free image to be referenced for scoring [13]. Traditional NR-IQA methods include the Blind Image Quality Index (BIQI) [14], the Blind/Referenceless Image Spatial Quality Evaluator (BRISQUE) [15], and the Natural Image Quality Evaluator (NIQE) [16]. With the development of deep learning in recent years, Kang et al. [17] first proposed an NR-IQA model using a Convolutional Neural Network (CNN) to extract image features. After that, some deep-learning-based NR-IQA models were also proposed [18,19]. Although the above IQA models have outstanding performance in the quality assessment of natural scene images, their application in view synthesis is very limited. This is because there are also local geometric distortions generated by the depth images and DIBR process in the view synthesis, which cannot be handled by the general IQA model [6].

In this case, view synthesis quality metrics have been proposed [20,21,22] to evaluate the image quality after DIBR-based view synthesis. However, the DIBR process is computationally expensive and time-consuming. For this reason, it is very meaningful and valuable to predict the image quality after view synthesis from pre-synthesis texture and depth images [23,24,25]. Nevertheless, current quality evaluation methods for view synthesis basically use hand-designed features. The performance improvement of traditional methods is relatively slow because of the shallow feature extraction of hand-designed methods [26]. In contrast, CNN has a strong expressive ability and is widely used in the field of quality evaluation of natural scene images [17]. Therefore, we consider applying deep learning in quality prediction for view synthesis.

This paper proposes a blind quality prediction model based on HEterogeneous DIstortion Perception (HEDIP), which predicts the image quality of view synthesis from pre-synthesis texture and depth images. The distortions of texture and depth images usually lead to traditional and geometric distortions [25], i.e., heterogeneous distortions, in the DIBR-synthesized images. To obtain more edge information, the proposed model is designed as a Two-Channel Convolutional Neural Network (TCCNN) structure, which can extract features in the image spatial and gradient domain, respectively. Among them, the edge features extracted by the gradient channel can effectively reflect the geometric distortions. Furthermore, to better describe the geometric distortions, we add a Contextual Multi-Level Feature Fusion (CMLFF) module, which can fuse shallow detail features and deep semantic features. At the input of the proposed HEDIP model, the texture and depth images are fused by Discrete Cosine Transform (DCT) [27] to imitate the distortions of DIBR-synthesized images. The fused images are then fed to the TCCNN to extract features in the spatial and gradient domains. Additionally, a fully connected layer linearly regresses the extracted features into a quality score. Considering the presence of non-uniform distortions in the view synthesis image [25], the ground-truth score of the source image cannot effectively represent the labels of each image patch during training. Therefore, we design a Heterogeneous Distortion Perception (HDP) module with the help of the classic BRISQUE [15] metric and combine it with the ground-truth score of the source image to provide effective training labels for each image patch. The advantage of the proposed HEDIP model is demonstrated through extensive experiments and comparisons. The contributions of this paper are as follows.

1. We propose a deep-learning-based blind quality prediction model for view synthesis, a two-channel convolutional neural network structure based on the spatial-gradient domain, which operates end-to-end via input texture and depth images.
2. A heterogeneous distortion perception module is designed to provide effective training labels for each image patch.
3. Extensive experiments on different databases show that our proposed model achieves state of the art.

## 2. Related Work

Existing view synthesis quality metrics basically adopt hand-designed methods to extract features. Tian et al. [20] proposed a NIQSV metric by quantifying the distortions of synthesized images based on morphological and edge operations. Furthermore, they also proposed NIQSV+ [21] metric on this basis to evaluate blurred regions, holes, and stretching distortions. Gu et al. [22] first generated reconstructed images using the autoregression (AR) model and then measured the geometric distortions based on the error between the AR-reconstructed image and the corresponding DIBR-synthesized image. In [28], a No-Reference Morphological Wavelet with Threshold (NR-MWT) metric first obtained high-frequency information based on morphological wavelet and then mapped the high-frequency information to the quality score. Gu et al. [29] reported a Multiscale Natural Scene Statistical analysis (MNSS) method, which inferred the image quality mainly based on the degree of self-similarity impairment and major structure degradation at different scales. Zhou et al. [30] addressed a blind view composite quality metric, which used Difference-of-Gaussian features to measure edge degradation and texture unnaturalness. Wang et al. [31] decomposed the DIBR-synthesized images by using discrete wavelet transform and then calculated the quality score of the synthesized image based on the geometric distortions and global sharpness of the low-frequency and high-frequency sub-bands. Recently, Li et al. [32] reported a view synthesis quality metric based on local Instance DEgradation and global Appearance (IDEA). This model used discrete orthogonal moments and superpixels to measure local and global distortions, respectively.

The above works are all about quality evaluation of the images after view synthesis. The DIBR-based view synthesis process includes the acquisition, compression, transmission, and decompression of texture and depth images, as well as deformation and rendering in the DIBR process. In practical applications, different types and degrees of distortions may occur in each link of view synthesis. Moreover, the DIBR process is computationally intensive and complex To avoid unnecessary distortions and calculations, it is worth considering predicting the quality of view synthesis based on texture and depth images, which can make the view synthesis system more flexible. Currently, only a few studies have investigated quality prediction for view synthesis. Wang et al. [23] advised a novel FR quality prediction model, which utilized the classic SSIM [8] method to compute two quality indication maps between distorted images and reference images for texture and depth. The overall quality is calculated based on the two quality indication maps. Shao et al. [24] recommended a High-Efficiency View Synthesis Quality Prediction (HEVSQP) method with the help of sparse representation. They first achieved Color-Involved View Synthesis Quality Prediction (CI-VSQP) and Depth-Involved View Synthesis Quality Prediction (DI-VSQP), and then predicted the quality score of the synthesized view through the metrics of CI-VSQP and DI-VSQP models. Li et al. [25] put forward a prediction model based on color-depth image fusion, which fused the input texture and depth images through wavelet transform to imitate the synthesized images. The statistical features of the fused images are then mapped to quality scores.

## 3. Materials and Methods

The proposed HEDIP is a deep learning model that can predict the image quality of view synthesis without reference. The texture and depth images before synthesis are fused through DCT, and then the spatial and gradient domain features of the fused image are extracted to predict the quality score. Notably, for the problem that local distortion causes image patches to have no valid training labels, the designed HDP module can provide effective training labels for each image patch with the help of the classic BRISQUE metric and the ground-truth score of the source image.

### 3.1. Image Preprocessing

In DIBR-based view synthesis, the distortions of texture and depth images generally lead to traditional and geometric distortions in the synthesized images [31]. Therefore, we fuse texture and depth images to imitate the distortions of DIBR-synthesized images. It is worth emphasizing that DCT transform and inverse transform are real-time and lossless, so we fuse texture and depth images through DCT transform. Among the DCT coefficients, the low-frequency coefficients mainly represent the information that changes gently in image intensity (brightness/grayscale), and the high-frequency coefficients mainly represent the detailed information of the image [33].The low-frequency coefficients may contain noise information, and the high-frequency coefficients may contain geometric distortion information, both of which will degrade image quality [34]. As a result, we keep the low-frequency coefficients of the texture image and averagely fuse the high-frequency coefficients of the texture and depth image. Then the fused image is obtained by inverse DCT transform. The distortions of the texture image are directly transferred to the fused image, while the distortions of the depth image destroy the edge information of the fused image.

**Figure 1:**
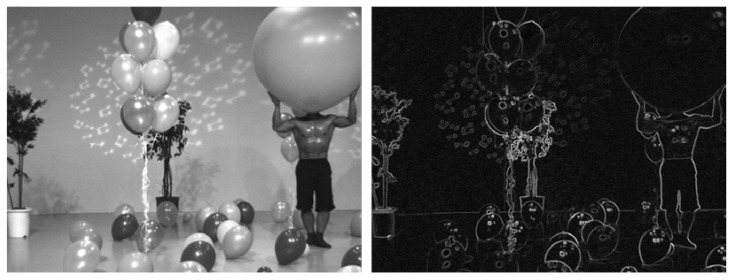
Fused image and corresponding gradient image.

Because the Sobel operator is fast and accurate in edge positioning, we choose to use the Sobel operator to calculate the gradient image. The gradient image $I_{g}$ of the spatial image $I_{d}$ is calculated as follows:

(1) $$I_{g}=\sqrt{G_{x}^{2}+G_{y}^{2}},$$

(2) $$G_{x}=M*I_{d},$$

(3) $$G_{y}=M^{T}*I_{d},$$

where $M=\left[\begin{array}{ccc} -1 & 0 & +1\\ -2 & 0 & +2\\ -1 & 0 & +1 \end{array}\right]$, T is the transpose operation, and * is the convolution operation. The fused image and the corresponding gradient image are shown in Figure 1. The gradient image can represent the edge information of the fused image well.

### 3.2. Two-Channel Convolutional Neural Network Structure

**Figure 2:**
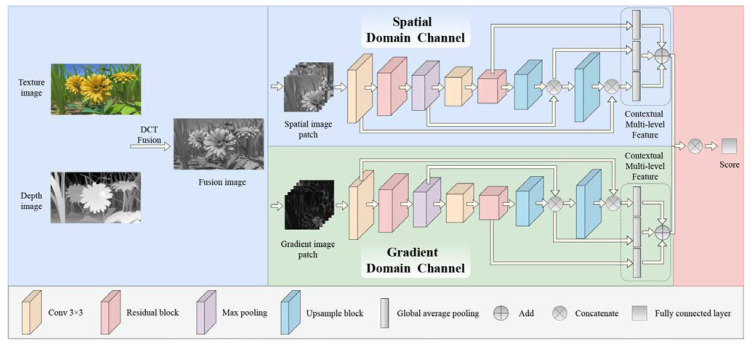
Two-channel convolutional neural network structure based on spatial-gradient domain.

**Figure 3:**
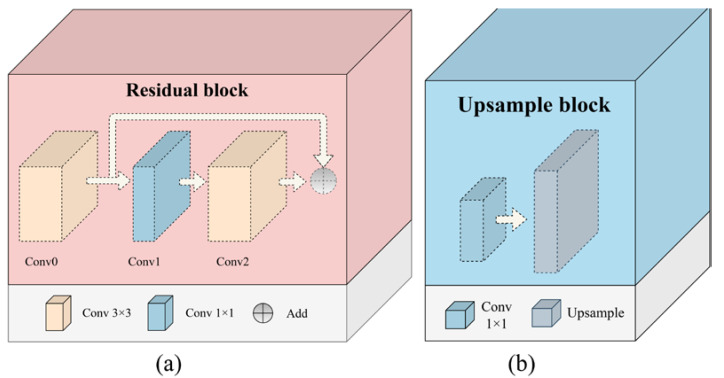
Residual block and Upsample block structure. (**a**) Residual block; (**b**) Upsample block.

To obtain more edge information, the proposed HEDIP model is designed as a Two-Channel Convolutional Neural Network structure, which can extract features in the image spatial and gradient domain, respectively. Among them, the edge features extracted by the gradient channel can effectively reflect the geometric distortions. The output of each layer in the proposed HEDIP model is shown in Table 1. To be specific, the network structure is shown in Figure 2, including Conv3 × 3, Residual block, Max pooling, Upsample block, Global average pooling, Add, Concatenate, and the Fully connected layer. Among them, the residual block can prevent gradient disappearance by reusing shallow features of the image. As shown in Figure 3a, the Residual block consists of Conv3 × 3, Conv1 × 1, and Conv3 × 3. Table 2 shows that the main function of Conv1 × 1 is to reduce the number of parameters. As shown in Figure 3b, the Upsample block is composed of Conv1 × 1 and Upsample. The function of Conv1 × 1 here is to change the number of channels, and the function of Upsample is to change the size of the deep features to match the shallow features. Notably, each convolutional layer is followed by a Rectified Linear Unit (ReLU) [35] activation function $z=max(0,\sum_{i}w_{i}a_{i})$, where z, $w_{i}$,and $a_{i}$ represent the output of the current layerand the weight and the output of the previous layer, respectively.

This paper denotes the spatial domain channel as $SDC\left(\cdot \right)$. The spatial domain feature is:

(4) $$F_{S}=SDC\left(w_{S},p_{S}\right),$$

where $w_{S}$ and $p_{S}$ denote the spatial domain channel weight and spatial image patch. Similar to the spatial domain channel, the gradient domain channel is denoted as $GDC\left(\cdot \right)$. The gradient domain feature is:

(5) $$F_{g}=GDC\left(w_{g},p_{g}\right),$$

where $w_{g}$ and $p_{g}$ represent the gradient domain channel weight and gradient image patch, respectively. Then, $F_{S}$ and $F_{g}$ are fused as:

(6) $$F=concat(F_{S},F_{g}),$$

where $concat\left(\cdot \right)$ represents the concatenating multiple features.

Finally, F is linearly regressed into the quality score by a fully connected layer.

### 3.3. Heterogeneous Distortion Perception Module

In DIBR-synthesized images, the overall distortion is different from the local distortion. From this point of view, the ground-truth score of the source image cannot be efficiently represented as the labels of each image patch during training.

**Figure 4:**
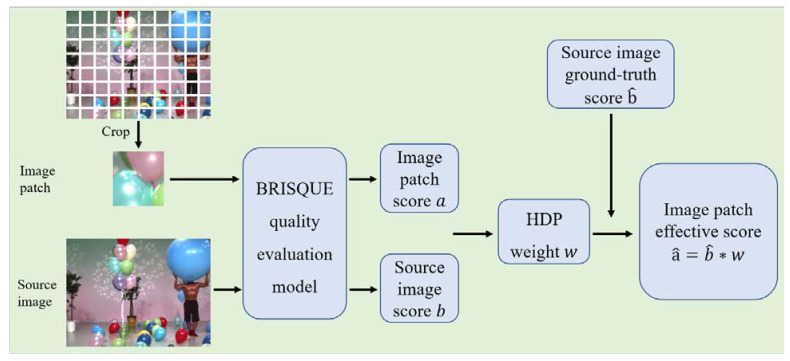
Heterogeneous distortion perception module.

To address this problem, we propose an HDP module, which is shown in Figure 4. The image patch and the corresponding source image are evaluated by the classic BRISQUE model to obtain scores a and b. Remarkably, unlike the ground-truth score of the source image, the evaluation standard of the BRISQUE model is that a large score corresponds to more serious distortion. If the quality of the image patch is lower relative to the quality of the source image, the score a of the image patch is larger than the score b of the source image. In this case, in order for the training label of the image patch to match the ground-truth score of the source image, i.e., the larger the score, the smaller the distortion, the HDP weight of the image patch is calculated as:

(7) $$w=\frac{b}{a},$$

where w represents the distortion of the image patch relative to the source image. When w is smaller, it indicates that the distortion of the image patch is more serious, and the corresponding score (training label) is smaller. Hence, the training label for the image patch can be computed as:

(8) $$\hat{a}=\hat{b}*w,$$

where $\hat{b}$ is the ground-truth score of the source image.

**Figure 5:**
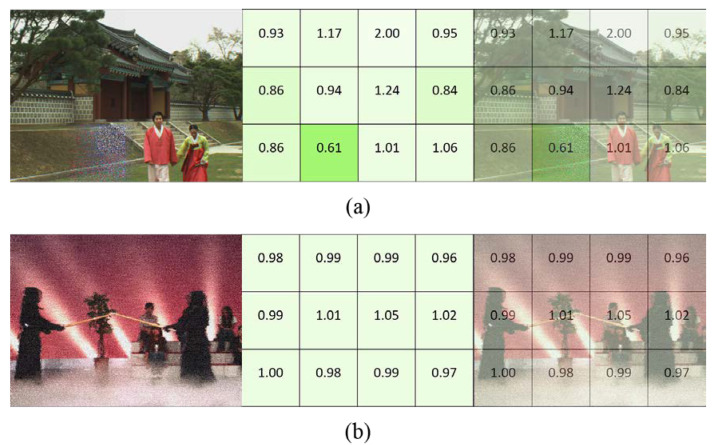
Visualization of HDP weights. The left image is a distorted image, the middle image is an HDP weight map, and the right image is an intuitive comparison of the distorted image and the HDP weight map: (**a**) case of local distortion; (**b**) case of global distortion.

Figure 5a shows the visualization of local distortion. It can be seen from the figure that the HDP weight w of the image patch with severe distortion is smaller, and the corresponding color is darker. A visualization of the global distortion is shown in Figure 5b, where the distortion perception weight w is almost the same for each image patch. The HDP module can be easily observed to be suitable not only for images with local distortion, but also for images with global distortion.

### 3.4. Contextual Multi-Level Feature Fusion Module

To better describe the geometric distortions, we propose a contextual multi-level feature fusion module, which fuses shallow detail features and deep semantic features. Figure 2 shows the module, and the feature names required for operation are shown in Table 1. First, the feature $F_{4\times 4}$(4 × 4 × 64) is adjusted to the $F_{4\times 4}^{\prime }$(16 × 16 × 32) through the Upsample block, and then we concatenate the $F_{4\times 4}^{\prime }$ and $F_{16\times 16}$(16 × 16 × 32) to obtain the $F_{16\times 16}^{\prime }$(16 × 16 × 64). In addition, the $F_{64\times 64}^{\prime }$(64 × 64 × 64) is obtained by operating the same steps as above for the $F_{16\times 16}^{\prime }$. Finally, the $GAP_{F64}$, $GAP_{F16}$, and $GAP_{F4}$ are obtained by global average pooling the $F_{64\times 64}^{\prime }$, $F_{16\times 16}^{\prime }$, and $F_{4\times 4}$, respectively. The weight of the *i*-th feature is recorded as:

(9) $$p_{i}^{*}=max\left(0,p_{i}\right)+\tau ,$$

where τ is a stable constant, which can guarantee $p_{i}^{*}>0$. Furthermore, the weights $p_{i}^{*}$ are normalized to:

(10) $$b_{i}=\frac{p_{i}^{*}}{\sum_{j}^{N_{p}}p_{j}^{*}},$$

where $N_{p}$ is equal to 3. Therefore, the feature F after fusion is calculated as:

(11) $$F=p_{1}^{*}GAP_{F64}+p_{2}^{*}GAP_{F16}+p_{3}^{*}GAP_{F4},$$

### 3.5. Training

We employ a window sliding strategy to divide the image into several 128×128 image patches to train our model. During the training phase, each image patch is provided with labels according to the designed HDP module. In the testing phase, the predicted score of the source image is obtained by averaging the predicted scores of all image patches in the source image. The mapping between extracted features and scores is achieved by minimizing the loss of predicted and ground-truth scores, so the loss function is designed as:

(12) $$min\;\frac{1}{N}\sum_{l=1}^{N}\|q_{l}-{\hat{q_{l}}}_{1}\|,$$

where N is the number of ttexture–depth image pairs in the training set, and $q_{l}$ and $\hat{q_{l}}$ denote the predicted score and training label of the i-th image patch, respectively. The proposed HEDIP model is implemented in Pytorch and runs on a Windows 10 system with a 3.70 GHz CPU and NVIDIA 2080 Ti GPU.

## 4. Experiments

### 4.1. Datasets and Evaluation Protocols

We conduct a series of experiments on the MCL-3D [36] and IST [37] databases to verify the performance of the proposed quality prediction metric for DIBR-based view synthesis. MCL-3D database [36].The database consists of 684 synthesized image pairs and corresponding Mean Opinion Score (MOS) value. Among them, 648 image pairs are generated by the View Synthesis Reference Software (VSRS) [36] using the ttexture–depth image pairs. There are three combinations of texture and depth images for view synthesis: (1) distorted texture images and undistorted depth images, (2) undistorted texture images and distorted depth images, and (3) distorted texture images and distorted depth images. Six kinds of distortions are applied to the input color and/or depth images, namely, Gaussian blur, JPEG compression, downsampling blurring, additive white noise, JPEG2000, and transmission error. IST database [37]. The database consists of 180 synthesized image pairs and corresponding MOS values. Among them, 120 image pairs are synthesized by the VSIM [38] algorithm, and the remaining 60 image pairs are synthesized by the VSRS [36] algorithm. Moreover, both the texture and depth images suffer from compression artifacts to varying degrees. It is worth noting that the images are synthesized by the VSIM and VSRS algorithms, respectively, in the DIBR-based view synthesis process. Therefore, for this database, we conduct two sets of experiments, respectively, on the texture and depth images required in the synthesis process of the VSIM and VSRS algorithms.

The MOS values of the synthesized images in the above two databases can be used as the ground-truth scores of input ttexture–depth image pairs. Furthermore, we adopt the Pearson Linear Correlation Coefficient (PLCC) and the Spearman Rank order Correlation Coefficient (SRCC) to evaluate model performance. PLCC is used to measure the performance of the model in terms of accuracy, and SRCC is used to measure the performance of the model in terms of monotonicity. The closer the PLCC and SRCC are to one, the better the model performance [24,39].

### 4.2. Performance Evaluation

We compare the proposed HEDIP model with state-of-the-art related models. Four general NR-IQA metrics are compared, namely, BRISQUE [15], NIQE [16], IL-NIQE [40], and M3 [41]. Quality evaluation metrics for view synthesis are compared, including MW-PSNR [42], MP-PSNR [43], LOGS [6], SET [30], Jakhetiya’s [44], and NIQSV [20]. In addition, the metric [23], which first proposed the idea of view synthesis quality prediction, is also compared. Depending on the scene, 80% of the image pairs are randomly selected for training, and the remaining 20% are used for testing. To avoid bias, the random split of the training test is repeated 10 times, and the average values are reported [45]. It should be noted that the metric [23] needs undistorted texture and depth images during the quality prediction, which are not provided in the IST dataset. Therefore, the PLCC and SRCC of the metric [23] on the IST database cannot be calculated.

The accuracy (PLCC) and monotonicity (SRCC) of the general quality evaluation, view synthesis quality evaluation, and view synthesis quality prediction models on the MCL-3D and IST databases are shown in Table 3, Table 4 and Table 5. The best result is highlighted in boldface, and the second best result is underlined. In Table 3, Table 4 and Table 5, ‘Post-DIBR’ indicates that the model uses DIBR synthesized images for quality evaluation, and ‘Pre-DIBR’ indicates that the model uses the texture and depth images to predict the quality of view synthesis. ‘GNR’ denotes the general no-reference quality metric and ‘VFR/VRR/VNR’ denotes the full-reference/reduced-reference/no-reference view synthesis quality metric. ‘T’ represents traditional methods, and ‘D’ represents deep learning methods. By comparison, it can be found from Table 3 that the proposed HEDIP model has the best performance in MCL-3D, in terms of both PLCC and SRCC. In addition, in terms of PLCC, the post-DIBR metric SET [30] has the second best performance. In terms of SRCC, the pre-DIBR metric [23] has the second best performance. For VSIM on the IST database (in Table 4), the HEDIP has the best PLCC as well as the second best SRCC. For VSRS on the IST database (in Table 5), the HEDIP delivers the best SRCC while also producing the second best PLCC (very close to the best SET [30]). In summary, the proposed HEDIP model achieves state-of-the-art overall performance. Moreover, as a pre-DIBR model, which is a deep learning model, the HEDIP outperforms the post-DIBR model.

**Figure 6:**
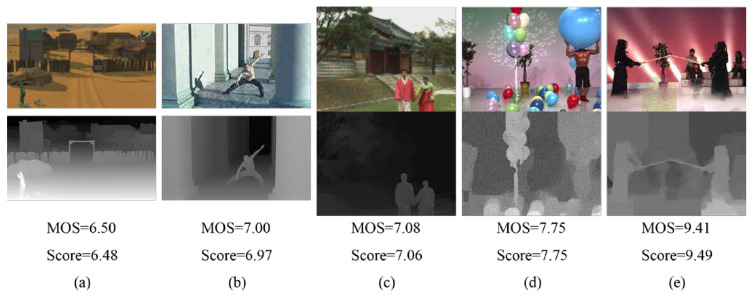
Texture–depth image pairs for six different scenes; the top row shows the texture image, and the bottom row shows the depth image. Each image pair comes with the MOS value and the predicted score. (**a**–**e**) are different scenarios.

To intuitively understand the performance of the proposed model, Figure 6 shows the ttexture–depth image pairs with different scenes and distortions, as well as the MOS values of the synthesized image and the predicted scores. From Figure 6a–e, it can be found that the predicted scores are very close to MOS values. Furthermore, when the MOS values increase, the predicted scores of the proposed model also increase. It can be seen that the prediction criteria of the proposed model are in line with the human scoring criteria.

### 4.3. Performance on Different Distortions

**Figure 7:**
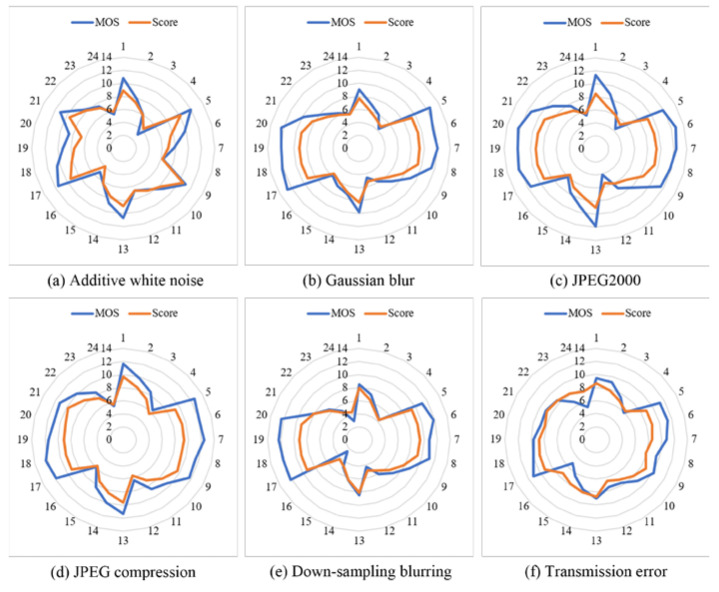
Radar plots of the MOS values and the predicted scores on the MCL-3D test set: (**a**) test result with additive white noise; (**b**) test result with Gaussian blur; (**c**) test result with JPEG2000; (**d**) test result with JPEG compression; (**e**) test result with downsampling blurring; (**f**) test result with transmission error.

**Figure 8:**
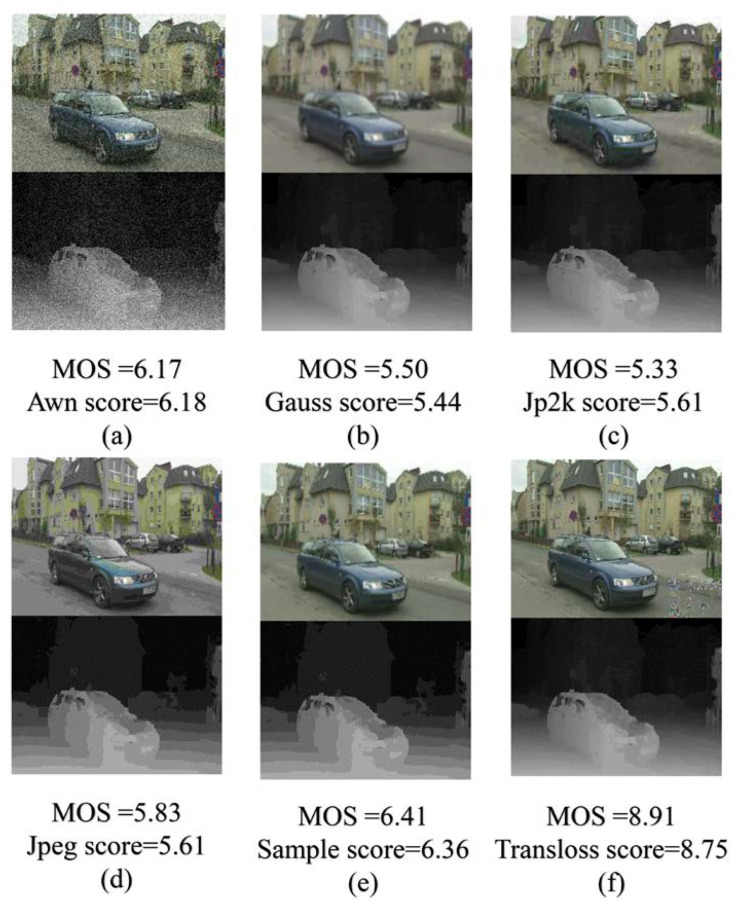
Texture–depth image pairs for six different distortions; each image pair comes with the MOS value and the predicted score: (**a**) additive white noise; (**b**) Gaussian blur; (**c**) JPEG2000; (**d**) JPEG compression; (**e**) downsampling blurring; (**f**) transmission error.

The MCL-3D database includes six distortion types. In order to investigate the performance of the proposed HEDIP model on different distortion types, we test images of six distortion types, respectively. Figure 7a–f show the radar plots of the proposed model with different distortion types on the MCL-3D test set; the blue line is the MOS value, and the orange line is the predicted score. The closer the two lines are, the more accurate the model is. On the other hand, the more similar the shapes are, the more monotonic the model is. It can be intuitively found from the radar plots that the HEDIP model still has excellent accuracy and monotonicity under different distortion types. Further, the MOS value for each distorted image pair is very close to the ground truth (given in Figure 8).

### 4.4. Ablation Study

To further demonstrate the effectiveness of the proposed modules, we conduct a series of ablation experiments based on the MCL-3D database, which use the same environment configuration as before. We use TCCNN without any modules as the base model. Then, the CMLFF module and the HDP module are added to the base model in turn for experiments.

**Figure 9:**
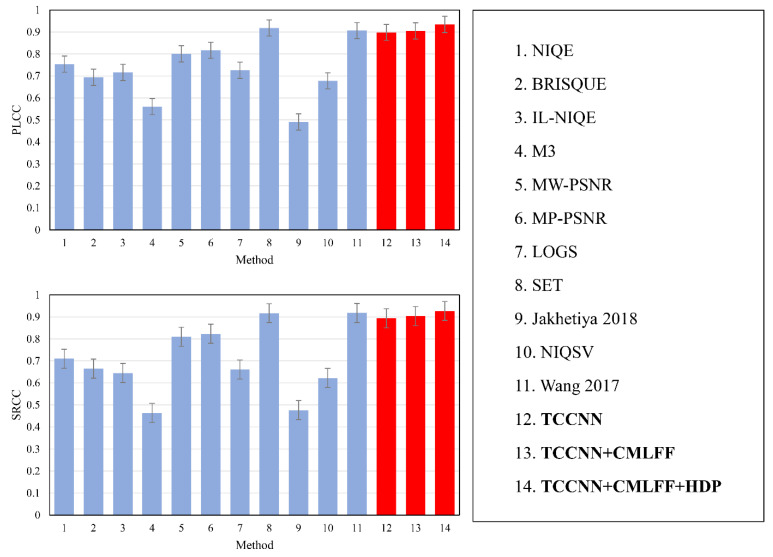
Performance and standard error bars of different methods on the MCL-3D database [23,44].

The experimental results are shown in Table 6. When CMLFF and HDP modules are added, the performance reaches the state of the art. From this result, we can see the importance and pertinence of each module. Moreover, it can be observed from Figure 9 that the basic TCCNN model outperforms most state-of-the-art view synthesis quality metrics.

## 5. Discussion

The current quality assessment methods for view synthesis basically use hand-designed features. Due to the shallow feature extraction of hand-designed methods, the performance improvement of traditional methods is relatively slow. Inspired by the above efforts, we proposed a blind quality prediction model based on heterogeneous distortion perception, which predicts the image quality of view synthesis from pre-synthesis texture and depth images. The proposed deep learning model is a two-channel architecture that can extract features in the spatial and gradient domains. Furthermore, due to the presence of local distortion in the view synthesis image, we address a heterogeneous distortion perception module to provide effective training labels for each image patch. The experimental results demonstrate the effectiveness of the proposed model.

The quality prediction model can make the view synthesis system more flexible, considering that if the input color/depth images cannot generate satisfactory synthesized viewpoint (by prediction), their quality can be adjusted before sending to the time-consuming DIBR process. The current quality assessment methods for view synthesis basically use hand-designed features, while convolutional neural networks can learn more effective features, which may promote the development of quality assessment technology for view synthesis. Although our model achieves very high performance in predicting the quality of view synthesis, we believe that further improvements to the backbone network in future work may still have the potential to improve the overall performance of the model. The work in this paper mainly evaluates the quality of view synthesis of images. With the demand for high-quality visuals, evaluating the view synthesis quality of videos is a very promising direction. Therefore, in following work, we may extend from the two-dimensional quality evaluation to the three-dimensional quality evaluation; of course, this will be challenging.

## 6. Conclusions

The quality of synthesized images affects the development and application of DIBR-based view synthesis technology. Most of the current view synthesis quality metrics evaluate the image quality after DIBR-based view synthesis and use hand-crafted methods to extract features. On the one hand, the DIBR process is computationally expensive. On the other hand, shallower hand-crafted features may affect the performance improvement. To tackle these problems, we have proposed a blind quality prediction model based on heterogeneous distortion perception, which predicts the image quality of view synthesis from pre-synthesis texture and depth images. To the best of our knowledge, the proposed model is the first to apply deep learning in the field of view synthesis quality assessment, while predicting the synthesized images without the complex DIBR process. The proposed model has been designed as a two-channel convolutional neural network structure, which can extract spatial and gradient domain features separately. Furthermore, we have designed a heterogeneous distortion perception module, which can provide effective training labels for image patches in source images. Extensive experiments have been conducted on two public view synthesis image databases. The experimental results have demonstrated the superior performance of the proposed model.

The work of this paper is to predict the image quality after view synthesis without DIBR-based view synthesis, which will make the view synthesis system more sensitive. If the predicted synthesis quality is low before synthesis, it can be adjusted in time to avoid complex calculations. In future work, improving the backbone network of the proposed model can optimize the quality prediction performance. Due to the strong ability of deep learning to learn features, the wider application of convolutional neural networks in the field of quality evaluation of view synthesis may promote the development of this field.

## Figures and Tables

**Table 1:** Information of each layer of the two-channel convolutional neural network structure.

Layer Name	Output Size
Spatial/Gradient image patch	128 × 128 × 1
Conv3 × 3/ReLU	64 × 64 × 32 ($F_{64\times 64}$)
Residual block	32 × 32 × 32
Max pooling	16 × 16 × 32 ($F_{16\times 16}$)
Conv3 × 3/ReLU	8 × 8 × 64
Residual block	4 × 4 × 64 ($F_{4\times 4}$)
Upsample block	16 × 16 × 32
Concatenate	16 × 16 × 64
Upsample block	64 × 64 × 32
Concatenate	64 × 64 × 64
Global average pooling 1/2/3	1 × 1 × 64
Add	1 × 1 × 64
Concatenate	1 × 1 × 128
Fully connected layer (Score)	1

**Table 2:** Information of each layer of the residual block.

Layer Name	Padding	Filter Size	Stride
Conv0/ReLU	1	3 × 3	2
Conv1/ReLU	0	1 × 1	1
Conv2/ReLU	1	3 × 3	1
Add	/	/	/

**Table 3:** Performances of view synthesis quality metrics on the MCL-3D and IST database. The best result is highlighted in boldface, and the second best result is underlined.

Category	Metric	Type	MCL-3D Database	
			PLCC	SRCC
Post-DIBR	NIQE [16]	GNR-T	0.754	0.710
	BRISQUE [15]	GNR-T	0.694	0.665
	IL-NIQE [40]	GNR-T	0.716	0.645
	M3 [41]	GNR-T	0.561	0.463
	MW-PSNR [42]	VRR-T	0.801	0.810
	MP-PSNR [43]	VRR-T	0.817	0.823
	LOGS [6]	VRR-T	0.726	0.661
	SET [30]	VNR-T	0.918	0.917
	Jakhetiya [44]	VNR-T	0.491	0.476
	NIQSV [20]	VNR-T	0.678	0.622
Pre-DIBR	Metric [23]	VFR-T	0.906	0.918
	**HEDIP**	VNR-D	**0.934**	**0.927**

**Table 4:** Performances of view synthesis quality metrics on the VSIM-based IST database. The best result is highlighted in boldface, and the second best result is underlined.

Category	Metric	Type	VSIM on IST Database	
			PLCC	SRCC
Post-DIBR	NIQE [16]	GNR-T	0.584	0.586
	BRISQUE [15]	GNR-T	0.651	0.588
	IL-NIQE [40]	GNR-T	0.396	0.379
	M3 [41]	GNR-T	0.662	0.612
	MW-PSNR [42]	VRR-T	0.684	0.677
	MP-PSNR [43]	VRR-T	0.722	0.727
	LOGS [6]	VRR-T	0.630	0.627
	SET [30]	VNR-T	0.815	**0.803**
	Jakhetiya [44]	VNR-T	0.357	0.367
	NIQSV [20]	VNR-T	0.377	0.359
Pre-DIBR	Metric [23]	VFR-T	/	/
	**HEDIP**	VNR-D	**0.866**	0.787

**Table 5:** Performances of view synthesis quality metrics on the VSRS-based IST database. The best result is highlighted in boldface, and the second best result is underlined.

Category	Metric	Type	VSRS on IST Database	
			PLCC	SRCC
Post-DIBR	NIQE [16]	GNR-T	0.640	0.620
	BRISQUE [15]	GNR-T	0.745	0.711
	IL-NIQE [40]	GNR-T	0.613	0.599
	M3 [41]	GNR-T	0.713	0.719
	MW-PSNR [42]	VRR-T	0.572	0.564
	MP-PSNR [43]	VRR-T	0.552	0.535
	LOGS [6]	VRR-T	0.634	0.608
	SET [30]	VNR-T	**0.753**	0.710
	Jakhetiya [44]	VNR-T	0.504	0.343
	NIQSV [20]	VNR-T	0.521	0.455
Pre-DIBR	Metric [23]	VFR-T	/	/
	**HEDIP**	VNR-D	0.750	**0.767**

**Table 6:** Ablation results on the MCL-3D database. The **HEDIP** is the model proposed in this paper.

Module	PLCC	SRCC
TCCNN	0.898	0.894
TCCNN + CMLFF	0.905	0.904
TCCNN + CMLFF + HDP (**HEDIP**)	0.934	0.927

## Data Availability

The used datasets were obtained from publically open source datasets from: 1. MCL-3D: http://mcl.usc.edu/mcl-3d-database/, (1 September 2022); 2. IST: https://github.com/jascenso/ISTSynthesizeDataset, (1 September 2022).

## Abbreviations

The following abbreviations are used in this paper.

DIBR	Depth-Image-Based Rendering
TCCNN	Two-Channel Convolutional Neural Network
CMLFF	Contextual Multi-Level Feature Fusion
HDP	Heterogeneous Distortion Perception
IQA	Image Quality Assessment
FR	Full-Reference
RR	Reduced-Reference
NR	No-Reference
DCT	Discrete Cosine Transform
CNN	Convolutional Neural Network
MOS	Mean Opinion Score
VSRS	View Synthesis Reference Software
PLCC	Pearson Linear Correlation Coefficient
SRCC	Spearman Rank order Correlation Coefficient
